# Two independent variants of epidermal growth factor receptor associated with risk of glioma in a Korean population

**DOI:** 10.1038/s41598-022-23217-6

**Published:** 2022-11-08

**Authors:** In Ki Baek, Hyun Sub Cheong, Seok Namgoong, Jeong-Hyun Kim, Seok-Gu Kang, Seon-Jin Yoon, Se Hoon Kim, Jong Hee Chang, Lyoung Hyo Kim, Hyoung Doo Shin

**Affiliations:** 1 Department of Life Science, Sogang University, Seoul, 04107 Republic of Korea; 2 Research Institute for Life Science, GW Vitek, Inc., Seoul, Republic of Korea; 3 Department of Medicine, University of Ulsan College of Medicine, Seoul, Republic of Korea; 4 Department of Neurosurgery, Yonsei University College of Medicine, Seoul, Republic of Korea; 5 Department of Biochemistry and Molecular Biology, College of Medicine, Yonsei University, Seoul, Republic of Korea; 6 Department of Pathology, Yonsei University College of Medicine, Seoul, Republic of Korea; 7 Research Institute for Basic Science, Sogang University, Seoul, Republic of Korea

**Keywords:** Genetic association study, Genome informatics

## Abstract

Gliomas are the most common primary tumors in the brain and spinal cord. In previous GWASs, SNPs in *epidermal growth factor receptor* (*EGFR*) have been reported as risk loci for gliomas. However, *EGFR* variants associated with gliomas in the Korean population remain unstudied. This study explored the association of *EGFR* SNPs with the risk of glioma. We genotyped 13 *EGFR* exon SNPs in a case–control study that included 324 Korean patients diagnosed with glioma and 480 population-based controls. Statistical analyses of the association between *EGFR* SNPs and glioma risk were conducted using logistic regression. Both stepwise analysis and conditional logistic analysis were performed to identify independent associations among genotyped variants. We confirmed that two SNPs (*rs2227983, rs1050171*) were significantly associated with glioma (*rs2227983*: odds ratio = 1.42, *P*^*corr*^ = 0.009; *rs1050171*: odds ratio = 1.68, *P*^*corr*^ = 0.005). Additionally, the stepwise analysis and conditional logistic analysis indicated that both SNPs created variants with independent genetic effects. This study is the first to show evidence that functional variants of *EGFR*, namely, *rs2227983* (K521R) and *rs1050171* (Q787Q), are associated with an increased risk of glioma in the Korean population. Future work should confirm the functional association between *EGFR* variants and glioma.

## Introduction

Gliomas are the most common primary brain and spinal tumors, representing 81% of malignant brain tumors. Gliomas occur in the brain and central nervous system (CNS) especially in glial or precursor cells^1,2^. In the 2007 World Health Organization (WHO) classification of tumors of the CNS, gliomas were classified according to their histological characteristics as Grade I–IV^3,4^. In the 2016 WHO classification, gliomas were classified according to molecular properties, such as *isocitrate dehydrogenase* (*IDH*) and 1p/19q status. According to its histological and molecular properties, a glioma is classified as a diffuse astrocytoma, anaplastic astrocytoma, oligodendroglioma, anaplastic oligodendroglioma, or glioblastoma (GBM)^1,5,6^.

Genome-wide association studies (GWASs) have been performed to identify regions associated with the risk of gliomas. Previous studies have reported variants at 27 loci associated with the risk of glioma^7–10^, these include, eight loci associated with all glioma (3q26.2, 5p15.33, 7p11.2, 8q24.21, 9p21.3, 11q23.3, 17p13.1, and 20q13.33), seven loci associated with GBM (1p31.3, 11q14.1, 12q23.3, 12q23.33, 16q12.1, 16p13.3, and 22q13.1), and 12 loci for non-GBM glioma (1q32.1, 1q44, 2q33.3, 3p14.1, 10q24.33, 10q25.2, 11q21, 11q23.2, 12q21.2, 14q12, 15q24.2, and 16q13.3).

*Epidermal growth factor receptor* (*EGFR*) is located at 7p11.2, and is essential for cell survival and development^11^. Many cancers, including glioma, are known to increase EGFR activity due to gene mutations, overexpression, or amplification.^12,13^. *EGFR* plays an especially key role in gliomas^12^. Several studies have shown that *EGFR* variants are associated with the risk of glioma. For example, *rs1468727* and *rs730437* are associated with an increased risk in the Han Chinese population^14,15^. Similarly, *rs2252586* and *rs11979158* are associated with an increased risk in the Caucasian population^11,16^. In a meta-analysis, *rs11506105* was associated with an increased risk in both Asian and Caucasian populations^17^. Previous studies have confirmed the association between common genetic variants of *EGFR* and the heritable risk of gliomas. However, the association between the risk of gliomas and *EGFR* SNPs has not been studied in Korean populations.

To examine this association, we first selected SNPs of EGFR. Due to the large number of *EGFR* variants (> 5500 variants), we only considered important coding variants and previous glioma variants. We also performed an association analysis between susceptibility alleles and glioma subgroups with respect to clinical characteristics such as grades and histological and molecular properties.

## Material and methods

### Study subjects

A total of 804 subjects that are 324 cases, and 480 controls was analyzed in this study. The sample of glioma patients (*n* = 324) were collected at the Yonsei University Severance Hospital and collaborating hospitals, diagnosed between 2006 and 2016. Case subjects were divided to glioma subgroups based on the histologic and molecular properties according to 2007 and 2016 WHO classification of CNS tumors^3,4^. Patients who had history of other cancers were excluded through clinical record review. The population control (PC) samples (*n* = 480), which excluded participants who had a past medical history of various cancer types, were provided by the National Biobank of Korea, the Korean Genome and Epidemiology Study (KoGES) Consortium^18^. The controls were composed of quality-controlled biospecimen collections from population-based cohorts which comprised 10,038 blood donors aged 40–60 years from the Ansung-Ansan Community-based Cohort in 2001. The institutional review board of Yonsei University Severance Hospital approved the study protocols and the patients gave written informed consent for participation. Genomic DNA was extracted from blood samples using the Wizard Genomic DNA Purification Kit (Promega, Madison, WI).

The molecular alterations (*IDH* mutation and 1p/19q codeletion) were assessed in the following methods at Yonsei University Severance Hospital^19^. They investigated the molecular profile of all patients, which included 1p/19q codeletion, O-6-methylguanine-DNA methyltransferase (*MGMT*) promoter methylation, and IDH mutation status. The *IDH* mutation status was initially evaluated using immunostaining for the *IDH1-R132H* mutation using a Ventana Bench Mark XT autostainer (Ventana Medical System, Inc., Tucson, AZ, USA) according to the protocol. The antibody used was anti-human IDH1 R132H mouse monoclonal antibody (Clone H09L, 1:80 dilution; Dianova, Hamburg, Germany). In the absence of a positive mutant IDH1-R132H with immunohistochemistry, sequencing of IDH1 codon 132 and IDH2 codon 172 was performed. FISH analysis of 1p/19q status was performed using the LSI 1p36/1q25 and 19q13/19p13 Dual-Color Probe Kit (Abbott Molecular Inc., Abbott Park, IL, USA). Acquired images were interpreted by an experienced neuropathologist as the basis for Euro-CNS protocols^20^. If the numbers of “deleted” nuclei exceed 50%, the tumor was considered to show a “deletion” for the targeted chromosome.

All procedures performed in studies involving human participants were in accordance with the ethical standards of the institutional and/or national research committee and with the 1964 Helsinki declaration and its later amendments or comparable ethical standards.

### SNP selection and genotyping

The candidate SNPs of *EGFR* were selected for genotyping from the Japanese and Han Chinese population in the 1000 genomes database with minor allele frequency (MAF) > 5%. The final 13 SNPs in *EGFR* were selected based on functional variants position and high linkage disequilibrium (LD) between SNPs interest (r^2^ > 0.98). Also, we included four SNPs (*rs11979158, rs2252586, rs11506105* and *rs1468727*) that previously were reported to have association with the risk of gliomas. The primer tool was designed for the Fludigm SNP Type™ (San Francisco, CA, USA) to detect candidate SNPs except for two SNPs (*rs17290169* and *rs56183713*) because of non-designable. In addition, genotyping was performed in all 804 subjects (324 cases and 480 controls) by using the Fludigm EP1 system (Fludigm 96.96 SNPtype™, San Francisco, CA, USA). The genotype data were analyzed with the BioMark SNP Genotyping analysis software (version 4.3.2). All candidate SNPs have been submitted to dbSNP (batch ID: EGFR_Glioma_SNP): https://www.ncbi.nlm.nih.gov/SNP/snp_viewTable.cgi?handle=GDLABSOGANGLF.

### Statistical analysis

Linkage disequilibrium (LD) analysis between genotyped SNPs was carried out using the haploview v4.2 software from the Broad Institute (http://www.broadinstitute.org/mpg/haploview). Each individual haplotypes were estimated using PHASE 2.1 software^21^. To analyze the association with *EGFR* variants, logistic regression analysis under additive model was used for calculating Odds ratios (ORs), 95% confidence intervals, and corresponding *P*-values by adjusting age and sex as covariates using Golden helix SVS8 software (Bozeman, MT, USA). Also, the genotypes distribution such as the minor allele frequency (MAF) and Hardy–Weinberg equilibrium (HWE) of each SNP was compared in glioma patients and PCs. The *P*-values were corrected by Bonferroni correction for multiple testing of 13 times. In addition, to identify independent association among the significant *EGFR* variants, stepwise analysis and conditional logistic analysis were conducted using Statistical Analysis System (SAS) 9.4 software (SAS Inc., Cary, NC, USA). Subsequently, referent model analysis based on the allele distribution of SNPs (*rs2227983* and *rs1050171*) was performed to verify detailed genetic effect using the Golden Helix SVS8 software (Bozeman, MT, USA). An in silico analysis was conducted for identifying function of associated SNPs using the SNPinfo (http://snpinfo.niehs.nih.gov/snpinfo/snpfunc.html).

### Ethical approval

All procedures performed in studies involving human participants were in accordance with the ethical standards of the institutional and/or national research committee and with the 1964 Helsinki declaration and its later amendments or comparable ethical standards.

### Informed consent

Informed consent was obtained from all individual participants included in the study.

## Results

### Subjects’ characteristics

Glioma patient cases (*n* = 324, mean age = 51.0 ± 14.8 years, 52.7% male) were classified according to histological characteristics into diffuse astrocytoma (*n* = 32, mean age = 46.3 ± 12.2 years, 53.1% male), anaplastic astrocytoma (*n* = 46, mean age = 41.9 ± 14.5 years, 47.8% male), oligodendroglioma (*n* = 16, mean age = 46.1 ± 7.2 years. 50.0% male), anaplastic oligodendroglioma (*n* = 22, mean age = 44.0 ± 10.7 years, 63.6% male) and GBM (*n* = 201, mean age = 55.4 ± 14.4 years, 52.7% male). According to the 2016 WHO classification of CNS tumors, of 324 glioma patients, IDH-mutants were found in 87 patients while 1p/19q codeletion were found in 68 patients. The population control group consisted of 480 individuals over the age of 40 years (mean age = 54.8 ± 9.5 years, 49.4% male). The detailed classifications of cases are summarized in Table 1.

**Table 1:** Clinical characteristics of study subjects.

Groups (WHO grade)	Number of subjects	Age (mean + SD)	Male (%)	Molecular alteration (n)	
				*IDH1 or IDH2 status* (mutant/wildtype/NOS)	1p/19q codeletion (yes/ no/ NOS)
Gliomas (II–IV)	324	51.0 ± 14.8	52.7%	87/230/7	68/241/15
Diffuse astrocytoma (II)	32	46.3 ± 12.2	53.1%	21/11/0	6/26/0
Anaplastic astrocytoma (III)	46	41.9 ± 14.5	47.8%	12/34/0	6/38/2
Oligodendroglioma (II)	16	46.1 ± 7.2	50.0%	14/0/2	16/0/0
Anaplastic oligodendroglioma (III)	22	44.0 ± 10.7	63.6%	21/0/1	22/0/0
Glioblastoma (IV)	201	55.4 ± 14.4	52.7%	16/185/0	17/175/9
NOS	7	39.6 ± 9.9	57.1%	3/0/4	1/2/4
Population controls	480	54.8 ± 9.5	49.4%	–	–

*SD* standard deviation, *IDH* isocitrate dehydrogenase, *NOS* not otherwise subclassified in glioma.

### Genotyping *EGFR* genetic variants

**Figure 1:**
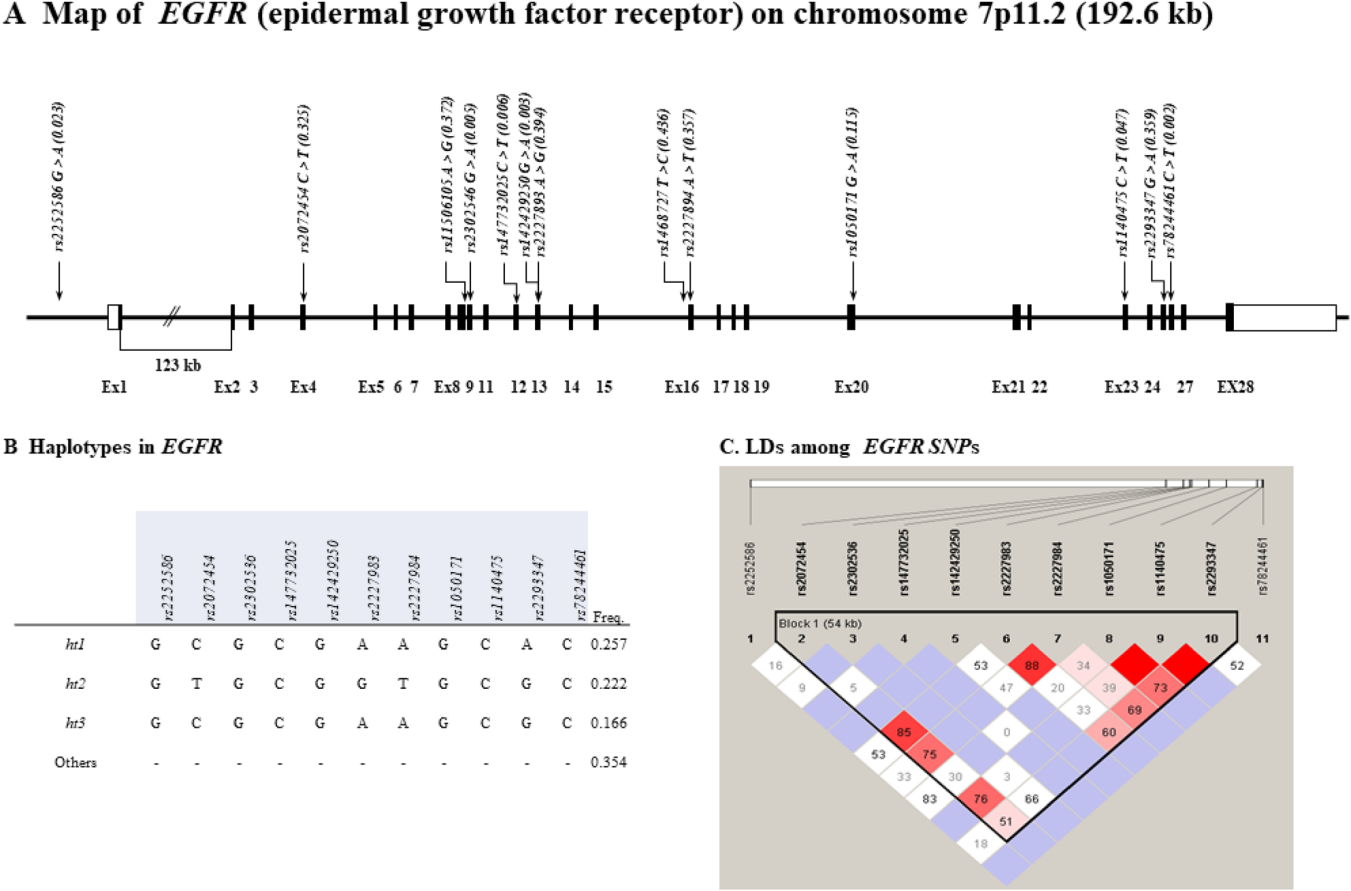
Physical map, haplotypes, and LDs of EGFR (epidermal growth factor receptor). (**A**) Physical map of EGFR and its SNPs genotyped in this study. Black blocks indicate coding exons; white blocks indicate 5'-untranslated region (UTR) and 3'-UTR. Score in the bracket indicates the minor allele frequency (MAF) of SNP. (**B**) Haplotypes of *EGFR*. Only common haplotypes with frequency over 0.05 are analyzed for association analyses. (**C**) LD plot of *EGFR*. SNPs investigated in this study compose one LD block. Number in block represents the value of LD coefficient │*D*'│.

A physical map of genotyped *EGFR* SNPs located on chromosome 7p11.2, is shown in Fig. 1A. One linkage disequilibrium (LD) block was constructed as shown in Fig. 1C. The LD block was composed of four haplotypes with a frequency > 5%, as shown in Fig. 1B. Additional information, such as SNP alleles, coordinates, and positions, is presented in Table 2.

**Table 2:** Genotyped *EGFR* SNP information and association of variants with risk of glioma.

SNP	Allele	Coordinate	Position (AA change)	MAF		HWE *P*		Additive		*P*^corrc^
				Glioma (n = 324)	PCs (n = 480)	Glioma (n = 324)	PCs (n = 480)	OR (95%CI)	*P*	
*rs2252586*^a^	G>A	54,978,924	5'UTR	0.008	0.023	0.89	0.61	**0.32 (0.12–0.86)**	**0.01**	NS
*rs2072454*^*b*^	C>T	55,214,348	Exon 4 (N158N)	0.384	0.325	0.68	0.59	**1.34 (1.08–1.66)**	**0.008**	NS
*rs11506105*^a^	A>G	55,220,177	Intron	0.377	0.372	0.81	0.54	1.04 (0.84–1.30)	0.698	NS
*rs2302536*	G>A	55,224,338	Exon 9 (P373P)	0.005	0.005	0.93	0.91	0.79 (0.18–3.40)	0.75	NS
*rs147732025*	C>T	55,227,971	Exon 12 (L480L)	0.002	0.006	0.98	0.89	0.27 (0.03–2.26)	0.16	NS
*rs142429250*	G>A	55,229,247	Exon 13 (P518P)	0.006	0.003	0.91	0.95	2.12 (0.46–9.78)	0.33	NS
*rs2227983*	A>G	55,229,255	Exon 13 (R521K)	0.481	0.394	0.42	0.79	**1.42 (1.16–1.74)**	**0.0007**	**0.009**
*rs1468727* ^a^	T>C	55,230,105	Intron	0.479	0.436	0.49	0.85	1.18 (0.96–1.45)	0.12	NS
*rs2227984*	A>T	55,238,874	Exon 16 (T629T)	0.422	0.357	0.99	0.37	**1.32 (1.07–1.62)**	**0.01**	NS
*rs1050171*	G>A	55,249,063	Exon 20 (Q787Q)	0.179	0.115	0.92	0.75	**1.68 (1.26–2.24)**	**0.0004**	**0.005**
*rs1140475*	C>T	55,266,417	Exon 23 (T903T)	0.050	0.047	0.35	0.96	1.05 (0.65–1.70)	0.83	NS
*rs2293347*	G>A	55,268,916	Exon 25 (D994D)	0.319	0.359	0.09	0.55	0.82 (0.67–1.02)	0.07	NS
*rs78244461*	C>T	55,269,456	Exon 26 (A1048V)	0.005	0.002	0.93	0.96	2.00 (0.33–12.14)	0.45	NS

Logistic regression analysis under additive model was used for calculating ORs and corresponding *P*-values for SNPs controlling age and sex as covariates.

*AA* amino acid, *MAF* minor allele frequency, *PC* population control, *HWE* Hardy–Weinberg equilibrium, *OR* odds ratio, *CI* confidence interval, *NS* not significant.

Significant associations are shown in bold face.

The major allele of each variant was used as reference.

^a^SNPs reported in previous study on glioma.

^b^In absolute LD with *rs730437* (r^2^ = 1 & D’ = 1) (SNiPA (https://snipa.helmholtz-muenchen.de/snipa3/)).

^c^Bonferroni-adjusted *P*-values by 13 SNP tests.

### Associations between *EGFR* SNPs and glioma risk

To identify causal variants among *EGFR* SNPs associated with the risk of glioma in a Korean population, a logistic regression analysis under an additive model adjusted for age and sex as covariates was performed as shown in Table 2. As a result, five SNPs *(rs2252486, rs2072454, rs2227983, rs2227984 and rs1050171*) were significantly associated with the risk of glioma. After applying the Bonferroni correction, the two SNPs *rs2227983* (*P*^*corr*^ = 0.009 in the additive model) and *rs1050171* (*P*^*corr*^ = 0.005 in the additive model) remained significantly associated with the risk of glioma. Furthermore, three haplotypes (frequency > 5%) were used for logistic regression analysis, which revealed that *EGFR*-ht3 (OR = 0.69, *P* = 0.01) was associated with a decreased risk of glioma. Additionally, *EGFR*-ht2 was associated with an increased risk of glioma (OR = 1.32, *P* = 0.02). Additional information is provided in Supplementary Table S1.

### Genetic effects of variants on glioma risk

**Figure 2:**
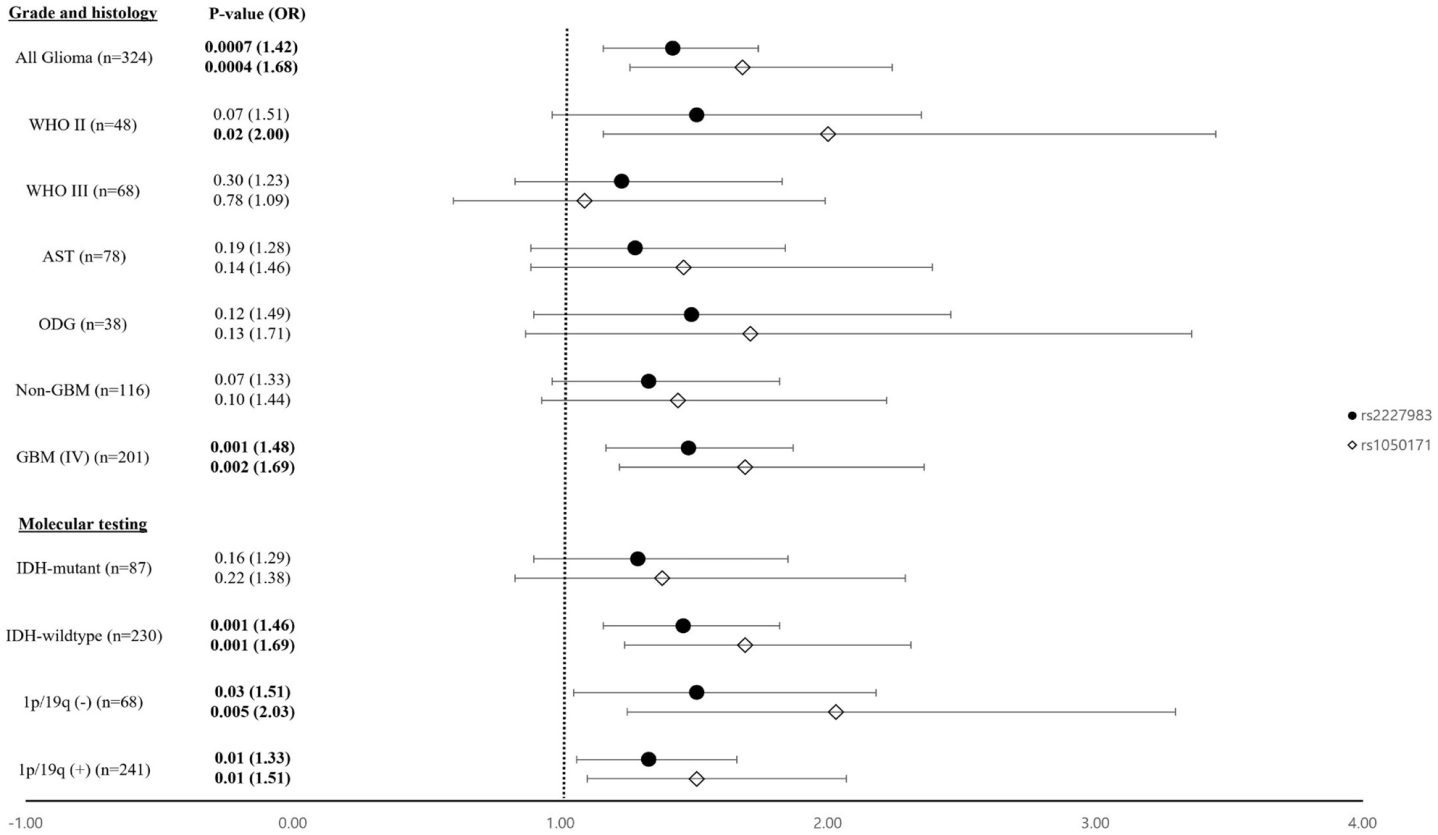
The association result of two independent SNPs between glioma subgroups and PCs. Logistic regression between glioma subgroups and PCs (n = 480) under additive model, adjusted by age and sex as covariates, was used for calculating ORs (95% CI) and P-values at rs2227983 and rs1050171. Each plot indicates the point estimate of ORs on the X-axis shown with 95% CI on the error bars. Significant associations are bolded. *PC* population control, *WHO* world health organization, *AST* astrocytomas, *ODG* oligodendrogliomas, *GBM* glioblastomas, *IDH-mutant* IDH1 or IDH2-mutated gliomas, *IDH-wildtype* IDH-wildtype gliomas, *1p/19q (-)* 1p/19q codeletion, *1p/19q ( +)* 1p/19q non-codeletion, *OR* odds ratio, *PCs* population control, *CI* confidence interval.

Stepwise and conditional analyses were performed on the two significant *EGFR* variants to verify the independent association between significant SNPs and glioma risk. In the stepwise analysis, two SNPs (*rs2227983 and rs1050171*) remained in the model at the parametric discriminant *P*-value (0.05). Subsequently, conditional logistic regression analysis indicated that the two SNPs were variants with independent genetic effects. The results of the two analyses are summarized in Table 3. The genetic effects of the two SNPs (*rs2227983 and rs1050171*) were then analyzed separately in the referent model. The GG genotype of *rs2227983* (OR = 2.07, 95% confidence interval [CI] 1.36—3.14) had a higher OR than the AG genotype (OR = 1.33, 95% CI 0.95–1.85) in referent analysis model (compared with AA referent groups) (Table 4). Thus, patients with two G alleles are likely to have a higher risk of glioma than patients with one G allele. Additionally, the AA genotype of *rs1050171* (OR = 2.60, 95% CI 0.96–7.01) had a higher OR than the GA genotype (OR = 1.71 95% CI 1.22–2.39) (Table 4) in referent analysis model. Thus, patients with two A alleles in *rs1050171* are likely to have a higher risk of glioma than patients with one A allele. Additionally, we investigated differences in the association between the two independent SNPs (*rs2227983 and rs1050171*) and glioma subgroups in relation to clinical characteristics such as WHO grade and, histological and molecular properties. These two variants were identified to be particularly associated with an increased risk of glioma in cases of GBM, IDH-wildtype and 1p/19q codeletion, as shown in Fig. 2.

**Table 3:** Independent association signals among glioma-associated *EGFR* variants.

SNP	*P*^corra^	Stepwise *P*^b^	Conditional *P*-value by	
			*rs2227983*	*rs1050171*
*rs2227983* (R521K)	**0.009**	**0.002**	–	**0.003**
*rs1050171* (Q787Q)	**0.005**	**0.0003**	**0.0009**	–

The *P*-values were obtained by logistic analysis between glioma patients (n = 324) and PCs (n = 480) under addictive model.

Significant associations are shown in bold face.

^a^Bonferroni-adjusted *P*-values by 13 SNP tests.

^b^The significance level was set at 0.05 in stepwise selection of glioma-associated *EGFR* SNPs.

**Table 4:** Logistic analysis of *rs2227983* and *rs1050171* in *EGFR* with the risk of Glioma.

SNPID	Genotype	Case, n(%)	PCs, n(%)	Referent		Additive		Dominant		Recessive	
				OR (95% CI)	*P*	OR (95% CI)	*P*	OR (95% CI)	*P*	OR (95% CI)	*P*
	AA	90 (28.0%)	176 (37.1%)	1							
*rs2227983* (R521K)	AG	153 (47.7%)	224 (47.2%)	1.33 (0.95–1.85)	0.09	1.42 (1.16–1.74)	**0.0007**	1.52 (1.11–2.07)	**0.008**	1.72 (1.20–2.46)	**0.003**
	GG	78 (24.3%)	75 (15.8%)	2.07 (1.36–3.14)	**0.0006**						
	GG	217 (67.4%)	377 (78.5%)	1							
*rs1050171* (Q787Q)	AG	95 (29.5%)	96 (20.0%)	1.71 (1.22–2.39)	**0.002**	1.68 (1.26–2.24)	**0.0004**	1.77 (1.28–2.45)	**0.0006**	2.26 (0.84–6.08)	0.10
	AA	10 (3.1%)	7 (1.5%)	2.60 (0.96–7.01)	0.06						

Logistic regression analysis under referent, additive, dominant, and recessive models are used for calculating ORs and corresponding P-values for SNP controlling age and sex as covariates.

The homozygotes of major allele were used as the referent group to the heterozygotes and homozygotes of the minor allele.

Significant values are in bold.

*PC* population control, *OR* odds ratio, *CI* confidence interval.

## Discussion

This study suggests that specific loci in *EGFR* are associated with an increased risk of glioma. Moreover, two independent coding variants (*rs2227983* and *rs1050171*) of gliomas were found in the Korean population. Additionally, we verified the association between *EGFR* coding variants and glioma subgroups based on histological characteristics and molecular properties by referring to previous studies ^5^. The ORs for all glioma subgroups were higher than 1, but *P* -values for some subgroups were not significant, as shown in Fig. 2.

A previous study indicated that *rs11979158* and *rs2252586* were significantly associated with gliomas in several European populations^11,22–24^. However, the association of these two SNPs with gliomas was not identified in Korean subjects. According to the 1000 Genomes database, the MAFs of *rs11979158* and *rs2252586* in the European populations (EUR) were 0.17 (*rs11979158*) and 0.28 (*rs2252586*). In our study, the MAFs of these SNPs in the Korean populations were 0.0006 (*rs11979158*) and 0.02 (*rs2252586*) (Supplementary Table S2). Thus, despite being reported as risk factors for glioma in Europeans, these SNPs were not risk factors for Korean glioma patients. The possible causal variants (*rs2227983* and *rs1050171*) in this study confirmed that the major and minor alleles between East Asian and European populations can differ based on the 1000 Genome Project (Supplementary Table S2). No studies have analyzed the association between these two variants and glioma in the European population. Consequently, glioma-associated genetic variants may vary by race or ethnicity, as allele frequencies differed by race (Supplementary Table S4).

The two SNPs (*rs1468727* and *rs11506105*) that were previously reported to be linked to glioma risk in other Asian populations were also analyzed^14,15^. No signals were detected with *rs11506105* or *rs1468727.* However, *rs2072454* (in absolute LD with *rs730437* in a Chinese population^14^) was significantly associated with glioma risk in our study (*P* = 0.008 before correction for multiple testing). However, considering the uncorrected *P* values in a study of Chinese population (*P* = 0.016 in the additive model) as well as this study (*P* = 0.008 in the additive model), these associations might be not reliable, as no statistical significance remained after correction in both studies.

EGFR is a cell membrane receptor that is activated by the binding of ligands such as EGF. Ligand binding to EGFR induces the activation of various signaling pathways, including the PI3K/AKT, Jak/Stat, JUNK, and MEK/ERK pathways, which can contribute to tumorigenesis. Variants in *EGFR* lead to overexpression of the EGFR protein have been associated with many cancers, including gliomas. Previous studies have reported that EGFR overexpression contributes to tumorigenesis and tumor progression in the classical subtype of gliomas^25,26^. According to Han et al. (2016), *rs2227983* is associated with the expression of *TP53* and p21 in Chinese hepatitis B virus-related hepatocellular carcinoma. In particular, the G allele has a higher p21 expression than the A allele. Additionally, according to their in silico analysis, *p21* and *EGFR* mRNAs were expressed in the same pathway or co-expressed^27^. In another in vitro experiment, the *rs2227983* variant (R521K, *P* = 0.0007 in this study) reduced EGFR ligand binding, growth stimulation, tyrosine kinase activation, and induction of proto-oncogenes^28,29^. This suggests that the *rs2227983* variant can increase EGFR activity through a substitution of the A allele with the G allele, leading to a change from lysine (K) to arginine (R). Moreover, this variant can induce overexpression of EGFR^30^, which can increase the risk of glioma^12,31^.

Previous studies have reported that *rs2227983* and *rs1050171* were associated with the risk of breast, lung, and colon cancer^32–34^, though one study found no association between *rs2227983* and the risk of lung cancer in Korean populations^35^. Other studies have shown that the *EGFR* 521R variant is associated with a poor prognosis in bladder cancer and colon cancer^36,37^. However, to date, no studies have reported the association of these variants with the risk of glioma. The *rs1050171* variant is associated with the risk of lung cancer in European and Korean populations^38,39^, and one study showed that this variant is associated with renal disease risk in the Korean population^40^. Although *rs1050171* is a synonymous mutation that does not substitute amino acids, it can affect mRNA stability or protein structure folding^41^. The variant *rs1050171* (G > A) is located in a highly conserved region, as predicted by SNPinfo (Supplementary Table S3). The synonymous variant *rs1050171* has a higher regulatory potential value (Reg potential = 0.489) than the nonsynonymous variant *rs2227983* (Reg potential = 0.390), shown in Supplementary Table S3. Moreover, *rs1050171* may affect *EGFR* gene expression and predispose patients to gliomas. Collectively, these two variants could increase the risk of gliomas by activating downstream signaling pathways through the overexpression of EGFR proteins*.*

We further investigated whether that two SNPs (*rs2227983* and *rs1050171*) are associated with brain tissue gene expression using eQTL calculators in the GTEx database (https://gtexportal.org/home/testyourown) ^42^. We found that the two variants were associated with gene expression in some brain tissues. This information is shown in Supplementary Table S5. However, no information was found regarding sQTLs.

Recently, advances in gene expression analysis, such as molecular profiling, have provided more predictive information than WHO classification of glioma^43^. Mutations in *IDH1* and *IDH2* have been frequently observed in astrocytoma and oligodendroglioma patients^44^. The 1p/19q codeletion is most common among oligodendroglioma patients and is used as a prognostic biomarker^43,45,46^. Oligodendroglioma patients also have both *IDH* mutations and 1p/19q codeletion in almost all cases, as shown in Table 1. In particular, *rs2227983* and *rs1050171* have a more significant association with *IDH*-wildtype subgroups than *IDH*-mutant subgroups. A previous study showed that primary GBM patients typically exhibit IDH-wildtype properties, obtained similar to the results in this study^47^ (shown in Table 1). These findings suggest that the risk of GBM is associated with belonging to *IDH*-wildtype subgroups. Additionally, the ORs of *rs1050171* were higher than those of *rs2227983* in almost all glioma subgroups except the WHO Grade III groups, as shown in Fig. 2. Because of limitations in statistical power, such as the low MAFs in 6 SNPs (MAF < 0.1) and small sample sizes, especially, in glioma subgroups analyses, interpretation of this study’s results requires caution. In this study, we used PCs matched for age and sex with insufficient clinical information, such as susceptibility to glioma, for detailed inclusion and exclusion criteria. Despite the use of these PCs, considering the difficulty of collecting large numbers of controls, this study can be considered as an alternative method to identify the genetic effects on gliomas^48^. Therefore, to determine the genetic effect of *rs2227983* and *rs1050171* on gliomas in a Korean population, subsequent clinical studies, such as mRNA and protein analyses, will be essential. In addition, although stepwise and conditional logistic analysis indicated two independent associations, it is not possible to know which SNP(s) are causal, because the causal variant(s) may be SNP(s) in LD with these SNPs. Further evidence from functional studies is needed to more confidently identify causal SNPs.

The purpose of this study was to investigate the genetic association between SNPs in *EGFR* and the risk of glioma in a Korean population. This study provided the first evidence that potentially functional polymorphisms in the *EGFR* gene, especially *rs2227983* (K521R) and *rs1050171* (Q787Q), may contribute to glioma susceptibility in the Korean population. Furthermore, it is essential for researchers in different populations to perform association studies of *EGFR* variants with glioma samples isolated from local population, as glioma-associated genetic variants may vary by ethnicity. This study will be useful for understanding and predicting the effect of SNPs on glioma susceptibility in Korean populations.

## Supplementary Information

Supplementary Information.

## Data Availability

The datasets generated during and/or analysed during the current study are available in the dbSNP repository, https://www.ncbi.nlm.nih.gov/SNP/snp_viewTable.cgi?handle=GDLABSOGANGLF. However, it has not been updated yet and will be publicly available when the databases release the next dbSNP Build (B156), which is planned for later this year.
