# Supplementary material for: Two independent variants of epidermal growth factor receptor associated with risk of glioma in a Korean population
Source: Sci Rep. 2022 Nov 8;12:19014. doi: 10.1038/s41598-022-23217-6 (PMC9643523; doi:10.1038/s41598-022-23217-6)
Supplement: Supplementary file 1 — Supplementary Information. [file 41598_2022_23217_MOESM1_ESM.docx]

| **Supplementary Table 1. Haplotype association analysis of *EGFR* with the risk of glioma** | | | | | | | | | | |
| --- | --- | --- | --- | --- | --- | --- | --- | --- | --- | --- |
| Haplotype | MAF | | HWE P-value | | Additive | | Dominant | | Recessive | |
|  | Glioma  (n=324) | PCs (n=480) | Glioma  (n=324) | PCs (n=480) | OR (95%CI) | *P* | OR (95%CI) | *P* | OR (95%CI) | *P* |
| ht1 | 0.239 | 0.269 | 0.291 | 0.70 | 0.83 (0.66-1.05) | 0.12 | 0.76 (0.57-1.01) | 0.06 | 0.99 (0.56-1.75) | 0.97 |
| ht2 | 0.255 | 0.206 | 0.771 | 0.66 | 1.32 (1.04-1.68) | **0.02** | 1.39 (1.04-1.86) | **0.03** | 1.52 (0.81-2.82) | 0.19 |
| ht3 | 0.134 | 0.186 | 0.131 | 0.09 | 0.69 (0.52-0.92) | **0.01** | 0.61 (0.44-0.84) | **0.002** | 1.24 (0.5-3.07) | 0.65 |

Logistic regression analysis under additive, dominant, and recessive models are used for calculating ORs and corresponding *P*-values for haplotypes controlling age and sex as covariates.

The groups that do not have each haplotype were used as referent subsets.

Significant associations are shown in bold face.

Abbreviation: MAF, minor allele frequency; PC, population control; HWE, Hardy-Weinberg equilibrium; OR, odds ratio; CI, confidence interval

| **Supplementary Table 2. Minor allele frequency of EGFR variants by populations** | | | | |  |
| --- | --- | --- | --- | --- | --- |
|  | **In this study (Korean)** | **CHB+JPT** | **EUR** | **AMR** | **study** |
| *rs2227983* (A>G) ^a^ | 0.43 | 0.46 | 0.73 | 0.67 | In this study |
| *rs1050171* (G>A) ^b^ | 0.14 | 0.14 | 0.60 | 0.55 | In this study |
| *rs11979158* (A>G) ^c^ | 0.0006 | 0.005 | 0.17 | 0.16 | Sanson et al. |
| *rs2252586* (C>T) ^c^ | 0.02 | 0.02 | 0.28 | 0.18 | Sanson et al. |

^a^ Minor allele of East Asian is G, but Minor allele of EUR and AMR is A, based on 1000 genome project.

^b^ Minor allele of East Asian is A, but Minor allele of EUR and AMR is G, based on 1000 genome project.

^c^ In GWAS and previous studies, these SNPs associated with glioblastomas in Americans.

Abbreviation: CHB, Chinese Han in Beijing; JPT, Japanese in Tokyo; EUR, European; AMR, American.

**Supplementary Table 3. Potential function of the two functional SNPs in EFGR gene as predicted by SNPinfo**

(http://snpinfo.niehs.nih.gov/snpinfo/snpfunc.html) software

| SNPID | Chromosome | Position^a^ | Allele | Splicing  (ESE or ESS) | nsSNP | Polyphen | RegPotential | Conservation |
| --- | --- | --- | --- | --- | --- | --- | --- | --- |
| rs2227983 | 7 | 55229255 | A/G | Y | Y | benign | 0.390 | 0.003 |
| rs1050171 | 7 | 55249063 | A/G | -- | -- | -- | 0.489 | 1 |

^a^ The position is based on NCBI GRCh37

SNP, single nucleotide polymorphism; EGFR, epidermal growth factor receptor; ESE, exon splicing enhancer; ESS, exon splicing silencer

nsSNP, non-synonymous single nucleotide polymorphism.

| **Supplementary Table 4. *P*-value in the Studies between EGFR variants and glioma.** | | | | | | | | | | |
| --- | --- | --- | --- | --- | --- | --- | --- | --- | --- | --- |
| **SNP** | **Allele** | **position** | **In this study** | **Sanson et al.** | **Melin et al.** | **Wibom et al.** | **Andersson et al.** | **Tian et al.** | **Hou et al.** | **Yan et al.** |
|  |  |  | **Korean** | **EUR+AMR** | **CEU** | **CEU** | **CEU** | **CHB** | **CHB** | **CHB** |
|  |  |  | **case: n=324**  **control: n=480** | **case: n=4146**  **control: n=7392** | **case: n=104**  **control: n=2868** | **case: n=598**  **control: n=595** | **case: n=607**  **control: n=1286** | **case: n=72**  **control: n=296** | **case: n=301**  **control: n=302** | **case: n=394**  **control: n=298** |
| *rs17172432* | T>C | Intron |  |  |  |  |  | 0.713 | 0.563 | 0.478 |
| *rs7809394* | C>T^a^ | Intron |  |  |  |  | **0.040** |  |  |  |
| *rs10225877* | A>T^a^ | Intron |  |  |  |  | **0.031** |  |  |  |
| *rs11979158* | A>G^a^ | Intron | ND | **7.03×10^-8^** | 0.21 | 0.374 |  |  |  |  |
| *rs917881* | G>A^a^ | Intron |  |  |  |  | **0.020** |  |  |  |
| *rs4947492* | A>G | Intron |  |  |  |  |  | 0.430 | 0.723 | 0.635 |
| *rs12718945* | G>T | Intron |  |  |  |  |  | 0.490 | 0.777 | 0.663 |
| *rs4947979* | A>G^a^ | Intron |  |  |  | 0.065 | **0.002** |  |  |  |
| *rs4947983* | A>C^a^ | Intron |  |  |  |  | **0.023** |  |  |  |
| *rs2072454* | C>T | N158N | **0.008** |  |  |  |  |  |  |  |
| *rs730437* | A>C | Intron |  |  |  |  | **0.032** | 0.575 | **0.016** | **0.010** |
| *rs11506105* | A>G^b^ | Intron | 0.698 |  |  |  | **0.012** | 0.794 | 0.053 | **0.042** |
| *rs4947986* | A>G^b^ | Intron |  |  |  | 0.091 | **0.044** |  |  |  |
| *rs2302536* | G>A | P373P | 0.75 |  |  |  |  |  |  |  |
| *rs147732025* | C>T | L480L | 0.16 |  |  |  |  |  |  |  |
| *rs142429250* | G>A | P518P | 0.33 |  |  |  |  |  |  |  |
| *rs2227983* | A>G^b^ | K521R | **0.0007** |  |  |  |  |  |  |  |
| *rs3752651* | T>C | Intron |  |  |  |  | **0.008** | 0.752 | 0.232 | 0.556 |
| *rs1468727* | T>C^c^ | Intron | 0.12 |  |  |  | **0.017** | 0.672 | **0.008** | **0.016** |
| *rs10228436* | A>G^b^ | Intron |  |  |  |  | **0.008** |  |  |  |
| *rs2227984* | A>T | T629T | **0.01** |  |  |  |  |  |  |  |
| *rs2252586* | A>G^b^ | - | **0.01** | **7.89×10^-8^** | 0.20 | 0.660 |  |  |  |  |
| *rs845552* | G>A^e^ | Intron |  |  |  |  | **0.009** | 0.175 | 0.105 | 0.062 |
| *rs9642393* | C>T^d^ | Intron |  |  |  |  | **0.031** | 0.092 | 0.115 |  |
| *rs1050171* | G>A^e^ | Q787Q | **0.0004** |  |  |  |  |  |  |  |
| *rs1140475* | C>T | T903T | 0.83 |  |  |  |  |  |  |  |
| *rs2293347* | G>A | D994D | 0.07 |  |  |  |  |  |  |  |
| *rs78244461* | C>T | A1048V | 0.45 |  |  |  |  |  |  |  |

^a^ MAF<0.05 in CHB and JPT based on 1000 genome project.

^b^ Minor allele of East Asian is G, but Minor allele of EUR and AMR is A, based on 1000 genome project.

^c^ Minor allele of East Asian is C, but Minor allele of EUR and AMR is T, based on 1000 genome project.

^d^ Minor allele of East Asian is T, but Minor allele of EUR and AMR is C, based on 1000 genome project.

^e^ Minor allele of East Asian is A, but Minor allele of EUR and AMR is G, based on 1000 genome project.

Abbreviation: OR, odds ratio; 95% CI, 95% confidence interval; ND: Not detected (MAF=0.0006 in Korean); CHB, Chinese Han in Beijing; JPT, Japanese in Tokyo; EUR, European; AMR, American; CEU, Northern Europeans from Utah.

Reference

1. Sanson M, Hosking FJ, Shete S, Zelenika D, Dobbins SE, et al. (2011) Chromosome 7p11.2 (EGFR) variation influences glioma risk. Hum Mol Genet 20: 2897-2904.
2. Melin B, Dahlin AM, Andersson U, Wang Z, Henriksson R, et al. (2013) Known glioma risk loci are associated with glioma with a family history of brain tumours -- a case-control gene association study. Int J Cancer 132: 2464-2468.
3. Wibom C, Spath F, Dahlin AM, Langseth H, Hovig E, et al. (2015) Investigation of established genetic risk variants for glioma in prediagnostic samples from a population-based nested case-control study. Cancer Epidemiol Biomarkers Prev 24: 810-816.
4. Andersson U, Schwartzbaum J, Wiklund F, Sjostrom S, Liu Y, et al. (2010) A comprehensive study of the association between the EGFR and ERBB2 genes and glioma risk. Acta Oncol 49: 767-775.
5. Jin TB, Li XL, Yang H, Jiri M, Shi XG, et al. (2013) Association of polymorphisms in FLT3, EGFR, ALOX5, and NEIL3 with glioblastoma in the Han Chinese population. Med Oncol 30: 718.
6. Hou WG, Ai WB, Bai XG, Dong HL, Li Z, et al. (2012) Genetic variation in the EGFR gene and the risk of glioma in a Chinese Han population. PLoS One 7: e37531.
7. Yan M, Li J, He N, Shi X, Du S, et al. (2017) A case-control study of the association between the EGFR gene and glioma risk in a Chinese Han population. Oncotarget 8: 59823-59830.

**Supplementary Table 5. Result of single-tissue expression quantitative trait loci (eQTL) (Data obtained from GTEx V8 portal, https://gtexportal.org/home/, GTEx Analysis Release V8 (dbGaP Accession phs000424.v8.p2))**

|  | | | | |
| --- | --- | --- | --- | --- |
| SNP | Gene Symbol | P-Value | NES | Tissue |
| rs2227983 | EGFR | 0.25 | -0.087 | Brain - Amygdala |
|  | EGFR | **0.014** | **-0.14** | **Brain - Anterior cingulate cortex (BA24)** |
|  | EGFR | **0.014** | **-0.12** | **Brain - Caudate (basal ganglia)** |
|  | EGFR | 0.78 | 0.011 | Brain - Cerebellar Hemisphere |
|  | EGFR | 0.71 | -0.015 | Brain - Cerebellum |
|  | EGFR | 0.33 | -0.046 | Brain - Cortex |
|  | EGFR | 0.27 | -0.043 | Brain - Frontal Cortex (BA9) |
|  | EGFR | 0.13 | -0.1 | Brain - Hippocampus |
|  | EGFR | 0.6 | -0.027 | Brain - Hypothalamus |
|  | EGFR | 1 | 0.000093 | Brain - Nucleus accumbens (basal ganglia) |
|  | EGFR | 0.44 | -0.038 | Brain - Putamen (basal ganglia) |
|  | EGFR | 0.99 | -0.00077 | Brain - Spinal cord (cervical c-1) |
|  | EGFR | 0.45 | -0.042 | Brain - Substantia nigra |
| rs1050171 | EGFR | 0.17 | -0.09 | Brain - Amygdala |
|  | EGFR | **0.036** | **-0.1** | **Brain - Anterior cingulate cortex (BA24)** |
|  | EGFR | 0.27 | -0.045 | Brain - Caudate (basal ganglia) |
|  | EGFR | 0.41 | -0.027 | Brain - Cerebellar Hemisphere |
|  | EGFR | 0.51 | -0.024 | Brain - Cerebellum |
|  | EGFR | 0.34 | -0.04 | Brain - Cortex |
|  | EGFR | **0.034** | **-0.081** | **Brain - Frontal Cortex (BA9)** |
|  | EGFR | 0.85 | 0.012 | Brain - Hippocampus |
|  | EGFR | 0.17 | -0.06 | Brain - Hypothalamus |
|  | EGFR | 0.5 | -0.024 | Brain - Nucleus accumbens (basal ganglia) |
|  | EGFR | 0.75 | -0.014 | Brain - Putamen (basal ganglia) |
|  | EGFR | 0.077 | -0.17 | Brain - Spinal cord (cervical c-1) |
|  | EGFR | 0.42 | 0.039 | Brain - Substantia nigra |
|  | EGFR-AS1 | 0.42 | -0.072 | Brain - Caudate (basal ganglia) |
|  | EGFR-AS1 | 0.38 | 0.088 | Brain - Cerebellum |
|  | EGFR-AS1 | 0.43 | -0.079 | Brain - Cortex |
|  | EGFR-AS1 | **0.00000014** | **-0.52** | **Brain - Spinal cord (cervical c-1)** |
|  | EGFR-AS1 | **0.0029** | **-0.37** | **Brain - Substantia nigra** |
| Significant associations (P < 0.05) are shown in bold face.  Abbreviations: NES: Normalized effect size | | |  |  |
